# Indigenous *Saccharomyces cerevisiae* Could Better Adapt to the Physicochemical Conditions and Natural Microbial Ecology of Prince Grape Must Compared with Commercial *Saccharomyces cerevisiae* FX10

**DOI:** 10.3390/molecules27206892

**Published:** 2022-10-14

**Authors:** Jie Gao, Mingfei Wang, Weidong Huang, Yilin You, Jicheng Zhan

**Affiliations:** 1 Beijing Key Laboratory of Viticulture and Enology, College of Food Science and Nutritional Engineering, China Agricultural University, Tsinghua East Road 17, Beijing 100083, China; 2 Beijing Chateau Lion Winery Co., Ltd., Beijing 102400, China; 3 College of Food Science and Nutritional Engineering, China Agricultural University, Tsinghua East Road 17, Beijing 100083, China

**Keywords:** indigenous *Saccharomyces cerevisiae*, fermentation, Prince grape, fungi, aroma

## Abstract

Indigenous *Saccharomyces cerevisiae*, as a new and useful tool, can be used in fermentation to enhance the aroma characteristic qualities of the wine-production region. In this study, we used indigenous *S. cerevisiae* L59 and commercial *S. cerevisiae* FX10 to ferment Prince (a new hybrid variety from Lion Winery) wine, detected the basic physicochemical parameters and the dynamic changes of fungal communities during fermentation, and analyzed the correlations between fungal communities and volatile compounds. The results showed that the indigenous *S. cerevisiae* L59 could quickly adapt to the specific physicochemical conditions and microbial ecology of the grape must, showing a strong potential for winemaking. Compared with commercial *S. cerevisiae* FX10, the wine fermented by indigenous *S. cerevisiae* L59 contained more glycerol and less organic acids, contributing to a rounder taste. The results of volatile compounds indicated that the indigenous *S. cerevisiae* L59 had a positive effect on adding rosy, honey, pineapple and other sweet aroma characteristics to the wine. Overall, the study we performed showed that selection of indigenous *S. cerevisiae* from the wine-producing region as a starter for wine fermentation is conducive to improving the aroma profile of wine and preserving the aroma of the grape variety.

## 1. Introduction

As a fermented alcoholic beverage, the quality of wine is affected by the microorganisms involved in fermentation. Wine fermentation is a complex microbial interaction process involving yeasts, bacteria and filamentous fungi. Throughout the fermentation process, changes in the diversity and abundance of microorganisms have an impact on the physicochemical properties and final aroma of wine [1,2]. Among them, yeasts are the core microorganisms in the wine fermentation process, in addition to completing the conversion of glucose and fructose in grape into ethanol and glycerol, they can also metabolize into many secondary metabolites such as alcohols, esters, acids and other secondary metabolites, which contribute to the formation of wine aroma and flavor [3]. The yeasts involved in wine fermentation include *Saccharomyces cerevisiae* and non-*Saccharomyces* yeasts composed of genera such as *Candida*, *Hanseniaspora*, *Pichia*, *Torulaspora*, and *Zygosaccharomyces*. Yeasts are present in the skin of the grapes, in the vineyard soil and the crushed grape must, and participate in the alcoholic fermentation along with the grapes [4]. Due to the low tolerance to ethanol and the sensitivity to low oxygen conditions, the number of non-*Saccharomyces* yeasts decreased rapidly with the progress of alcoholic fermentation, and *S. cerevisiae* dominates the alcoholic fermentation [5,6]. *S. cerevisiae* involved in fermentation plays a pivotal role in the quality of wines.

Commercial *S. cerevisiae* is often chosen in wine production to ensure the smooth start and completion of wine fermentation. The use of commercial *S. cerevisiae* can also reduce unpredictable flavor variations in the final product, lessen the risk of spoilage caused by spoilage strains, and result in wines of stable quality [7]. However, commercial *S. cerevisiae* cannot fully display the typical aroma of each grape variety, and its widespread use makes the sensory characteristics of each production region weaken, and the unique style cannot be exhibited, resulting in the phenomenon of homogenization of wines in each production region [8,9]. In addition, indigenous *S. cerevisiae* resources are abundant in wine regions. Excessive use of commercial *S. cerevisiae* as a starter ignores the plentiful wild yeast resources in the production region, and the limitations of using commercial *S. cerevisiae* gradually emerge. As a new biological tool, indigenous *S. cerevisiae* meets the needs of the wine market in terms of displaying the regional characteristics of the production region. Many winemakers prefer to choose indigenous *S. cerevisiae* as a starter for wine fermentation. This is because the indigenous *S. cerevisiae* isolated from grapes has good adaptability to the physicochemical conditions and microbial ecology of the grape must, and can more quickly coordinate and regulate the natural flora in the grape must [10,11,12]. At the same time, it is more conducive to highlighting the unique wine terroir characteristics of the production region and presenting the typical style characteristics of the production region in the wine [13].

In this study, we aimed to investigate the perturbation of the natural fungal community in grape must and the effect on flavor when commercial and indigenous *S. cerevisiae* were used in wine fermentation, respectively. To address this issue, we separately inoculated commercial and indigenous *S. cerevisiae* for Prince wine fermentation in winery. The Prince grape is a hybrid wine grape variety (Chambourcin x Viognier) bred by Lion Winery, with dark purple skin. The indigenous *S. cerevisiae* strain L59, isolated from the Lion Winery, was used as the starter in laboratory-scale fermentations and proved to have excellent wine-fermenting ability, which in turn could be used in the pilot scale fermentation. We used high-performance liquid chromatography (HPLC) to determine the basic physicochemical parameters in the samples during the fermentation process, and used headspace solid-phase microextraction (HS-SPME) combined with gas chromatography-mass spectrometry (GC-MS) to determine the concentration of volatile compounds in the samples to analyze the differences in aroma of the two groups of wines. The Illumina-based next-generation sequencing method was used to analyze the dynamic changes of fungi during fermentation, and to understand the perturbation patterns of the natural fungal community during fermentation by two *S. cerevisiae*. In addition, the correlation between fungi and volatile compounds was analyzed to reveal the potential impact of microorganisms on wine aroma. Our work helps to understand the impact of different *S. cerevisiae* on the perturbation of natural fungal community and aroma during wine fermentation, and provides theoretical guidance for the development and industrial application of indigenous *S. cerevisiae*.

## 2. Results

### 2.1. Interdelta Sequencing Typing of S. cerevisiae

**Figure 1:**
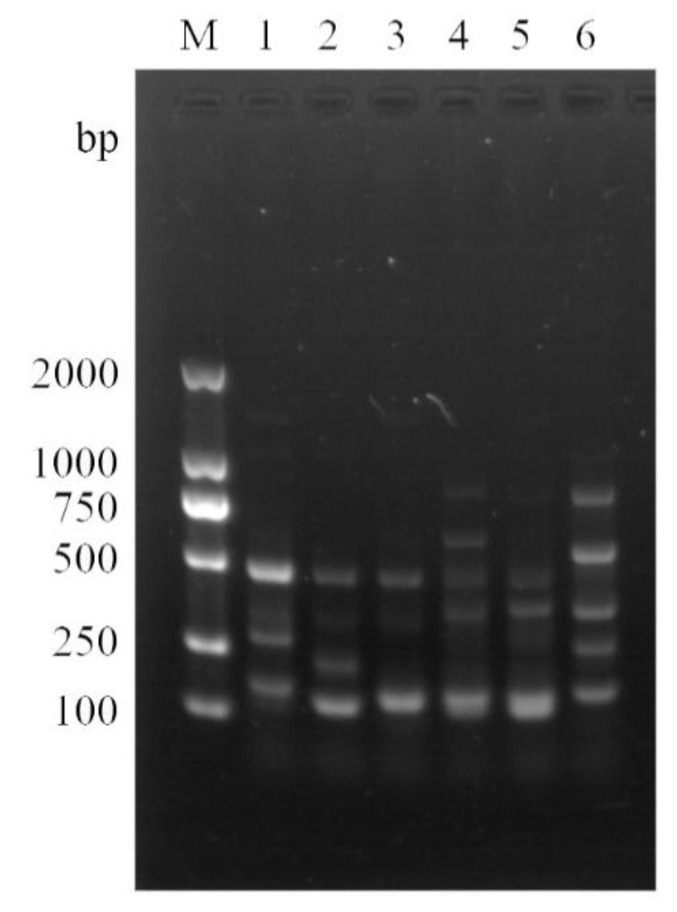
Gel electrophoresis images of different *S. cerevisiae* strains. M, D2000 DNA Ladder (Solarbio Life Science); 1, Safoeno NDA 21 (Fermentis); 2, Enartis Ferm ES488 (Enartis); 3, Enartis Ferm Red Vintage (Enartis); 4, Enartis Ferm AMR-1 (Enartis); 5, Zymaflore FX10 (Laffort); 6, L59.

Figure 1 showed the interdelta sequencing typing of five commercial *S. cerevisiae* strains and the indigenous *S. cerevisiae* L59 strain isolated from the winery. By interdelta sequence typing, all the analyzed strains were completely differentiated, and 6 different band types were identified. The results showed that the 6 strains of *S. cerevisiae* were strain-specific and that strain L59 was not identical to the commercial *S. cerevisiae* FX10 used by the winery.

### 2.2. Changes in Physicochemical Parameters during Fermentation

**Figure 2:**
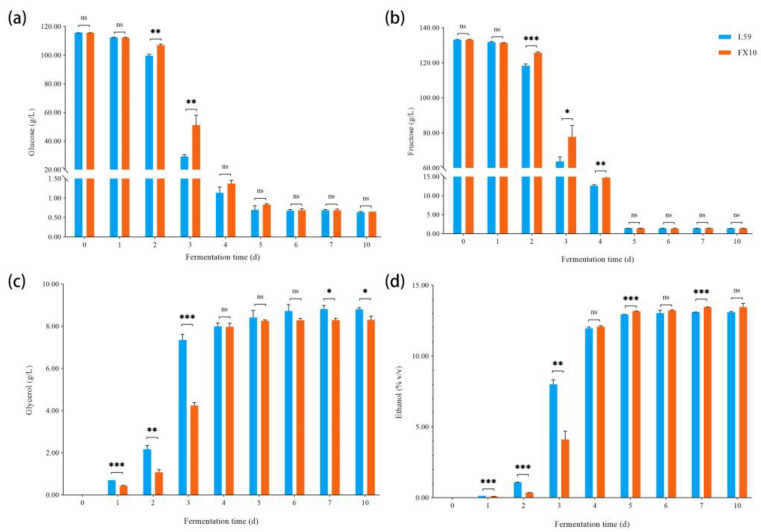
Changes of physicochemical indexes during the fermentation of the two groups. (**a**) Glucose; (**b**) Fructose; (**c**) Glycerol; (**d**) Ethanol. Note: *p*-values are indicated with asterisks (*). * 0.01 < *p* < 0.05, ** 0.001 ≤ *p* < 0.01, *** *p* < 0.001; ns indicates no significance.

The changes in physicochemical parameters of Prince grape must and samples at different fermentation stages were detected (Figure 2 and Table 1). Changes in total sugar, glycerol, and ethanol concentrations during fermentation can be used to monitor the fermentation process of wine, and can also reflect the state of microorganisms in the fermentation must [14]. The total sugar content of Prince grape mush was 248.78 g/L. As fermentation was in progress, glucose and fructose were converted by microorganisms into glycerol, ethanol and other metabolites. After the completion of alcoholic fermentation, the final total sugar contents of L59 and FX10 wines were 2.06 ± 0.02 g/L and 2.09 ± 0.05 g/L, glycerol contents were 8.81 ± 0.06 g/L and 8.31 ± 0.14 g/L, and ethanol contents were 13.10 ± 0.04% (*v*/*v*) and 13.46 ± 0.21% (*v*/*v*). By comparing the measurement results of physicochemical parameters in samples at different fermentation stages, it can be seen that although the fermentation rate of yeast L59 was faster than that of yeast FX10, the total sugar consumption and ethanol production of the two yeasts were similar, and there was no difference. However, the glycerol production of the L59 group was significantly higher than that of the FX10 group, which could offer a rounder taste to the wine.

Organic acids are important compounds in wine, which have a direct impact on the flavor and taste of wine [15]. In this study, the concentrations of 6 organic acids in Prince grape must and wine were detected (Table 1). After alcoholic fermentation, the tartaric and malic acid concentrations in the wine were significantly reduced. The tartaric acid content decreased from 7.36 ± 0.09 g/L to 4.73 ± 0.08 g/L (L59) and 5.09 ± 0.10 g/L (FX10). This was attributed to the decrease in the solubility of bitartrate and the precipitation of crystals as the alcohol concentration increases, thereby reducing the concentration of tartaric acid in the wine [16,17]. Malic acid content decreased from 1.43 ± 0.01 g/L to 1.02 ± 0.01 g/L (L59) and 1.25 ± 0.02 g/L (FX10). Compared with grape must, citric acid, succinic acid and acetic acid, typical products of alcoholic fermentation, were significantly increased in wine. In addition, 0.73 ± 0.01 g/L and 0.73 ± 0.02 g/L of lactic acid were detected in the two groups of wine samples, respectively, with no significant difference between the groups.

### 2.3. Changes in Volatile Compounds during Fermentation

**Figure 3:**
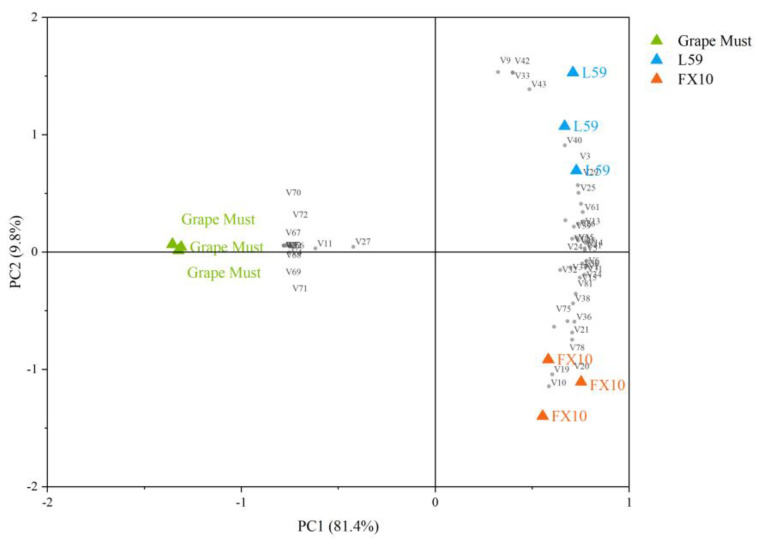
Principal component analysis (PCA) biplots of volatile compounds in Prince grape must and wines. Note: Volatile compound codes are provided in Table S1.

A total of 83 volatile compounds were detected from grape must and wine samples at different fermentation stages (Table S1), including 18 alcohols, 5 acids, 42 esters, 9 aldehydes and ketones, 6 olefins and 3 phenols. We analyzed these volatile compounds to better understand the dynamic changes of aroma components during fermentation (Figure S1). From the changes of volatile compounds, it can be seen that the aroma compounds in the wine fermentation process could be mainly divided into two categories. The first category was the aldehydes and ketones and C6 compounds that dominate in grape must and early stages of fermentation. The second category was the main volatile compounds in wine, including alcohols, esters and volatile acids. They can add flavors such as fruit, flowers and cream to wines and play a key role in the aroma profile of wines [18,19]. In this study, the contents of these three kinds of compounds increased significantly at the end of fermentation. Principal component analysis (PCA) was performed on 50 volatile compounds detected in grape must and two wines to more intuitively show the differences between volatile compounds in different samples. As shown in Figure 3, the variance contribution rate of the first principal component (PC1) was 81.4%, the variance contribution rate of the second principal component (PC2) was 9.8%, and the cumulative variance contribution rate reached 91.2%, which could explain most of the information on original data. The must and the wine fermented by two *S. cerevisiae*, respectively, were clearly separated. The compounds in the positive semi-axis region of the first principal component were mainly compounds associated with fermented aroma, such as phenethyl alcohol, octanol, ethyl caprylate, ethyl caprate, ethyl laurate, ethyl palmitate, and hexyl acetate. The negative semi-axis region of the first principal component included benzaldehyde, S-linalool oxide, β-damascone, 2-nonanone and C6 compounds, which had a strong negative correlation with the samples in the first principal component score. Therefore, the first principal component can be used to explain the aroma characteristics of fermentation in samples, which can clearly distinguish grape must and wines. In the positive semi-axis region of the second principal component, volatile compounds that have a strong positive correlation with the sample’s score in the second principal component included ethyl caprylate, ethyl caprate, benzyl alcohol, 3-ethoxy-1-propanol, 2,3-butanediol, 3-methylbutyl octanoate, methyl salicylate, etc., which brought rose fragrance, honey-like sweetness and fruit aromas such as pineapple to L59 fermented wine. The negative semi-axis region of the second principal component includes compounds such as isoamyl acetate, hexyl acetate, hexyl alcohol, 3-methylthiopropanol, and acids such as isobutyric acid. These compounds brought aromas of banana, coconut and other tropical fruit to FX10 wine, while acids also bring rancid flavors such as fatty and cheese to the wine. In addition, both L59 fermented wine and grape must were located on the positive semi-axis of the second principal component, indicating that the volatile aroma characteristics of the two groups were similar. The results showed that different *S. cerevisiae* altered the overall aroma quality of the wines, but the indigenous *S. cerevisiae* L59 could better preserve the varietal characteristics of Prince grapes.

### 2.4. Fungal Community Diversity and Richness Analysis

**Figure 4:**
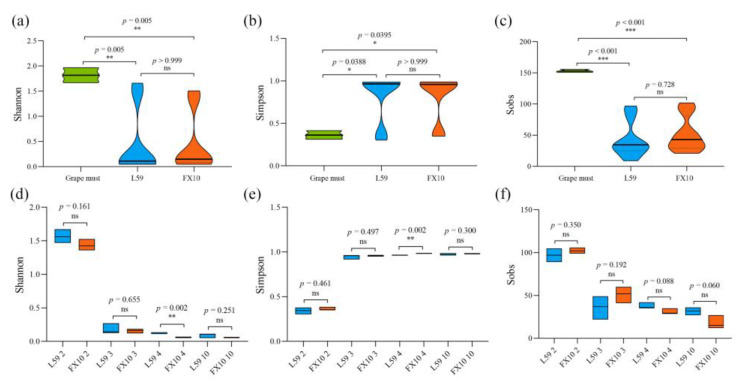
Alpha-diversity of the microbial community during the fermentation of Prince wine. Shannon index (**a**), Simpson index (**b**) and Sobs index (**c**) of grape must and two groups of fermentation samples; Shannon index (**d**), Simpson index (**e**) and Sobs index of samples at different fermentation stages (**f**). Note: *p*-values are indicated with asterisks (*). * 0.01 < *p* < 0.05, ** 0.001 ≤ *p* < 0.01, *** *p* < 0.001; ns indicates no significance.

The succession of the fungal community during the fermentation of Prince was investigated using high-throughput sequencing (HTS) technology. There was a total of 1,123,364 fungal gene sequences (391 bp in average length) clustered into 277 OTUs at the 97% sequence similarity level. Alpha diversity is a comprehensive indicator that can reflect the community richness and community diversity within a sample (Table S2) [20]. The Shannon index and the Simpson index are indices used to estimate the microbial diversity in a sample. The Sobs index is the actual number of observed OTUs reflecting microbial richness at a certain sampling level. We compared fungal community diversity and richness in grape must and samples from two fermentation groups (including samples on days 2, 3, 4, and 10). The results showed that the fungal community diversity (Figure 4a,b) and richness (Figure 4c) in the grape must were significantly higher than those in the fermented samples. The fungal community diversity (Figure 4d,e) and richness (Figure 4f) of the two groups of wine samples at different fermentation stages were comparatively analyzed. We could find that the diversity and richness of the fungal community showed a significant decrease as the fermentation proceeded (*p* < 0.05). According to the Shannon index and Simpson index, the fungal community diversity in the L59 group was significantly higher than that in the FX10 group on the 4th day of fermentation.

### 2.5. Dynamic Changes of the Fungal Community during Fermentation

**Figure 5:**
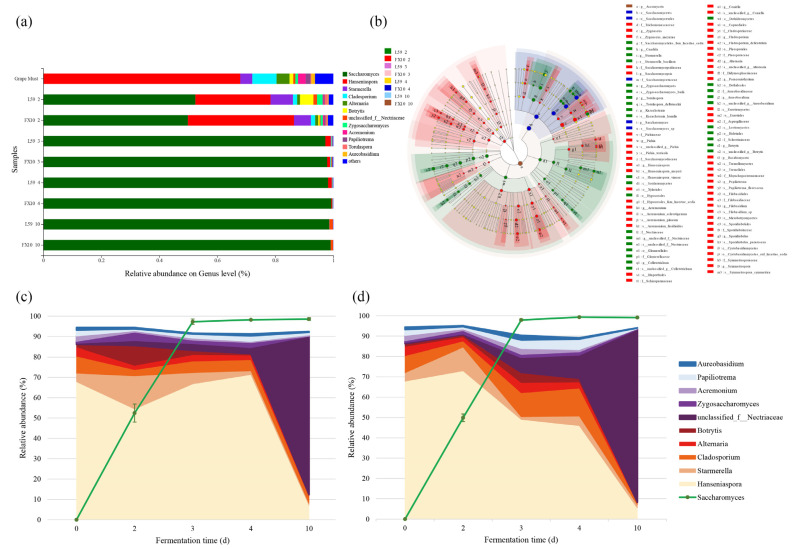
Changes in fungal community composition during the Prince wine fermentation. (**a**) Relative abundance of fungi at the genus level in samples at different fermentation times. (**b**) Linear discriminant analysis with effect size (LEfSe) analysis of microbial relative abundance data during fermentation (LDA > 3, *p* < 0.05). Changes in the relative abundance of fungal communities in fermentation samples inoculated with strain L59 (**c**) and yeast FX10 (**d**). Stacked area graphs show the relative abundance composition of the communities except *S. cerevisiae*.

The HTS results revealed the dynamic changes in the fungal community during the fermentation process, and the distribution of fungal genera with relative abundance greater than 1% was shown in Figure 5a. In Prince grape must, the most abundant genus was *Hanseniaspora*. In addition, fungal genera such as *Starmerella*, *Cladosporium* and *Alternaria*, which were common in Chinese wine-producing regions, were also detected in grape must [21]. With the progress of wine fermentation, the two groups of *S. cerevisiae* rapidly proliferated and dominated the alcoholic fermentation. On the 4th day of fermentation, the relative abundance of *S. cerevisiae* in the FX10 group was significantly higher than that in the L59 group. As the abundance of *S. cerevisiae* increased, the abundance of other taxa decreased, and the relative abundance of the fungal community showed significant changes.

Linear discriminant analysis (LDA) effect size (LEfSe) was used to analyze the differences in the abundance of fungal communities at different taxonomic levels from phylum to species in different fermentation stages between the two groups. LDA scores greater than 3.0 and *p* < 0.05 were used as criteria. Figure 5b showed all the taxonomy of each species that affect the differences between groups. The results showed that a total of 91 fungal taxa contributed to the differences among samples at different fermentation stages. In different fermentation stages, the abundance of non-*Saccharomyces* yeast population in the samples on fermentation day 2 was significantly higher than that in other stages. Among them, the abundance of *Zygosaccharomyces, Aureobasidium, Kazachstania, Candida, Colletotrichum, Starmerella, Torulaspora, Botrytis, and unclassified_f__Nectriaceae* in the second-day fermentation samples of L59 group was significantly higher than that of other fermentation stages; *Zygoascus, Saccharomycopsis, Alternaria, Cladosporium, Acremonium, Hanseniaspora, Pichia, Filobasidium, Papiliotrema, Coniella, Sporidiobolus, Paraconiothyrium, Symmetrospora* were significantly enriched in the second-day fermentation samples of the FX10 group. These also illustrated that the abundance of non-*Saccharomyces* yeast population decreased significantly as the alcoholic fermentation proceeded.

The relative abundance of the top 10 non-*Saccharomyces* genera was calculated by dividing the reads per taxon by the total reads per sample excluding *S. cerevisiae*. It could be observed that with the proliferation of *S. cerevisiae*, the non-*Saccharomyces* yeast populations also showed different patterns (Figure 5c,d). During the fermentation process, the relative abundance of *Hanseniasporas*, the dominant community in the non-*Saccharomyces* yeasts, first decreased, then increased, and then decreased again in the L59 group. In the FX10 group, the relative abundance of *Hanseniasporas* increased and then decreased. The *Zygosaccharomyces* has the same variation pattern as the *Hanseniasporas*. The dynamics of the *Botrytis* were different from theirs. The relative abundance of *Botrytis* increased and then decreased in the L59 group, while in the FX10 group it first decreased, then increased, and finally decreased again. Along with the change of non-*Saccharomyces* yeast populations, their relative abundances also showed significant differences (*p* < 0.05). On the second day of fermentation, the relative abundance of *unclassified_f__Nectriaceae* was significantly higher in the L59 group compared to the FX10 group. On day 4 of fermentation, the relative abundances of *Hanseniaspora*, *Alternaria* and *Zygosaccharomyces* in the L59 group were significantly higher than those in the FX10 group. Additionally, at the end of alcoholic fermentation (day 10), the relative abundances of *Hanseniaspora*, *Cladosporium*, and *Acremonium* were still significantly higher in the L59 group than in FX10. The results showed that the use of different *S. cerevisiae* had a significant effect on the dynamic changes in the microbial community throughout the fermentation process.

### 2.6. Correlation Analysis between Microbiota and Volatile Compounds

**Figure 6:**
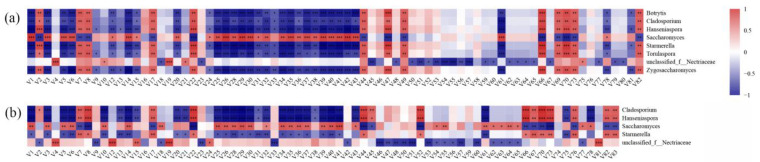
Spearman’s correlation analysis between microorganisms and volatile compounds during fermentation. (**a**) Core fungi in L59 group; (**b**) Core fungi in FX10 group. Note: Positive correlations on the heatmap are shown in brick-red and negative correlations are shown in navy-blue. Volatile compound codes are provided in Table S1. *p*-values are indicated with asterisks (*). * 0.01 < *p* < 0.05, ** 0.001 ≤ *p* < 0.01, *** *p* < 0.001; ns indicates no significance.

We explored the potential association of fungal taxa with the synthesis of volatile compounds during fermentation using Spearman’s correlation analysis and created a correlation heatmap with correlation coefficients between functional core fungi and volatile compounds. The core fungal communities associated with volatile compounds during the fermentation of Prince wines were shown in Figure 6 and were selected based on the following criteria: (i) The microbial communities were present throughout the fermentation process. (ii) O2PLS-DA model was constructed with fungal taxa as the independent variable and volatile compounds as the dependent variable, VIP (variable importance for predictive components) score >1.0 (Figure S2) [22] (iii) The absolute value of the Spearman’s correlation coefficient (R) between fungal communities and volatile compounds was >0.6 and *p* < 0.05 [23].

According to the above criteria, a total of 8 microbial genera were identified as the core microorganisms in the fermentation process. Among them, *Cladosporium*, *Hanseniaspora*, *Saccharomyces*, *Starmerella*, and unclassified_f__*Nectriaceae* were identified as core microorganisms in both groups. Furthermore, *Botrytis*, *Torulaspora*, and *Zygosaccharomyces* were identified as core microorganisms only in the L59 group. It is worth noting that the core microbial community may not directly produce relevant volatile compounds, but indirectly affect the concentration of volatile compounds in wine by affecting the synthesis or metabolism of the overall microbial community [24]. Compared with the FX10 group, there were more core microorganisms in the L59 group, which indicated that more fungal genera in the L59 group had an important impact on the changes of wine volatile compounds.

For wine, *Saccharomyces* is the main factor affecting the quality of wine, and it is also the main producer of alcohols and esters in wine [25]. In this study, *Saccharomyces* was significantly associated with multiple volatile compounds, especially alcohols and esters with significant positive associations (*p* < 0.05), but the correlation pattern of yeast was also different in the two groups. *Saccharomyces* was significantly negatively correlated with methyl hexanoate and isopentyl formate in the L59 group, and significantly positively correlated with methyl octanoate, but not significantly correlated with these three compounds in the FX10 group. In the FX10 group, *Saccharomyces* was significantly positively correlated with 3-methylthiopropanol and ethyl nonanoate, and significantly negatively correlated with phenethyl acetate. However, there were no significant correlations between *S. cerevisiae* and these three compounds in the L59 group. The non-*Saccharomyces* yeasts commonly present in wine fermentation, *Hanseniaspora* and *Starmerella*, showed a significant negative correlation with most alcohols and esters. However, they also showed a significant positive correlation with 2-hexen-1-ol, leaf alcohol, and diisobutyl phthalate. On the other hand, they also showed different correlations with the compounds in the two groups. *Hanseniaspora* and *Starmerella* showed a significant negative correlation with 1-pentanol, methyl salicylate, ethyl octadecanoate, and 2,4-di-tert-butylphenol in the L59 group. In FX10, however, there was no significant correlation with these compounds. *Hanseniaspora* and *Starmerella* in the L59 group did not show a correlation with diisobutyl adipate and 2-octanone, but in the FX10 group showed a significant positive correlation with these two compounds. As for unclassified_f__*Nectriaceae*, it showed a completely different correlation pattern. For example, it was significantly positively correlated with hexyl alcohol, isobutyric acid, and significantly negatively correlated with methyl n-caprate, methyl laurate, ethyl caproate, while other core microorganisms had no significant correlation with these compounds. In addition, in the L59 group, 3-methylthiopropanol was significantly positively correlated with unclassified_f__*Nectriaceae*, but not with other core microorganisms. In contrast, in the FX10 group, 3-methylthiopropanol was not significantly correlated with unclassified_f__*Nectriaceae*, but was significantly correlated with the other 4 core microorganisms. *Botrytis, Torulaspora, and Zygosaccharomyces*, which were only present in L59, were also closely related to the production of volatile compounds. The three genera, except for the inconsistent correlation of the four compounds, had the same correlation with other volatile compounds. *Torulaspora* and *Zygosaccharomyces* were significantly negatively correlated with benzyl alcohol, while *Botrytis* had no significant correlation with this compound. *Zygosaccharomyces* showed a significant negative correlation with 3-ethoxy-1-propanol, while the other two genera showed no significant correlation. *Torulaspora* had no significant correlation with dibutyl phthalate and 2,4-di-tert-butylphenol, while *Botrytis* and *Zygosaccharomyces* had a significant negative correlation with them. Overall, the microbial taxa and volatile compounds correlation patterns in Prince wine were not completely consistent.

## 3. Discussion

Aroma is an important part of wine quality evaluation, and it is also an important indicator to reflect the style of wine production regions. The terroir is the innate condition that determines the aroma of wine, and the microorganisms related to wine fermentation are the acquired factors that affect the aroma and flavor of the wine. Wine fermentation is a complex microbial interaction process involving the participation of multiple native microorganisms or inoculated strains. Microorganisms enter the winemaking system together with grapes or grape juice, and interact with each other to affect the aroma, flavor and quality of the wine. Previous studies have shown that the presence of different fungal populations on the surface of grape berries and in the early stage of fermentation is an extremely important microbial fingerprint for the flavor and quality of the wine [26,27]. The main fungal communities in Cannonau grape must in the Sardinia region of Italy were *Aureobasidium*, *Alternaria* and *Hanseniaspora* [28]. For Marselan grape must, the dominant fungal communities in the Huailai wine-producing region of China were *Aureobasidium* and *Alternaria* [21]. We analyzed the fungal genera in Prince grape must, and the results showed that *Hanseniaspora* and *Cladosporium* were the dominant fungal genera in the grape must. However, in wine fermentation, yeasts play a leading role, and among them, *S. cerevisiae* is the most critical microorganism in the wine fermentation process [29,30]. On the one hand, it is the main promoter of wine alcoholic fermentation, which can convert most of the sugar in grape must into ethanol and carbon dioxide. On the other hand, it maintains the position of dominant microorganisms in the fermentation process, which can largely avoid the infection of spoilage microorganisms to produce undesirable volatile compounds and fermentation stagnation, and is more controllable and less risky [31,32]. In wineries, inoculation of commercial *S. cerevisiae* is often used to ensure the successful completion of wine fermentation. However, inoculation of commercial *S. cerevisiae* for wine fermentation results in wines that are similar in flavor and aroma, has no regional characteristics, and also reduce the diversity of microorganisms during the fermentation process [33,34]. In recent years, many researchers and winemakers have shown an increased interest in indigenous yeasts in order to produce wines that can exhibit the qualities characteristic of wine regions. They tried to use indigenous *S. cerevisiae* that can adapt to the special habitat and microbial ecology of grape growing conditions, the special habitat of the grape must, and the microbial ecology, to select a starter that can adapt to and get along with natural microorganisms [8,12]. In this study, we selected indigenous *S. cerevisiae* L59 and commercial active dry yeast FX10 for pilot-scale fermentation. At the early stage of fermentation, the contents of glucose and fructose in the fermentation samples inoculated with yeast L59 were significantly lower than those of the FX10 group, and the concentrations of glycerol and ethanol in the L59 group were significantly higher than those of the FX10 group. This indicated that indigenous *S. cerevisiae* was significantly more dominant in the early stage of fermentation than commercial S. cerevisiae. Similarly, the research results of Capece et al. [35] also proved this point. We also observed that there were differences in the dynamics of microbial composition during fermentation in the two groups. Additionally, the results of the α-diversity analysis of fungi in Prince wine showed that although the fungal community diversity in samples was significantly reduced after inoculation with *S. cerevisiae*, on the fourth day of fermentation, the α-diversity of fungi in the group inoculated with yeast L59 was significantly higher than FX10 group. These may be due to the differences in the implantation capacity of the two *S. cerevisiae*, their ability to dominate the natural microflora present in the grape must, and their ability to compete with the natural microflora [8,12]. The indigenous *S. cerevisiae* L59 can get along more friendly with the natural microflora in grape must, while the commercial *S. cerevisiae* FX10 has the ability to predominate and maintain the dominance on the natural microflora in the fermentation process. These results confirmed that indigenous *S. cerevisiae* was better able to adapt to the physicochemical conditions in grape must, regulate the natural fungal community in grape must, and perform wine fermentation.

The chemical changes in wine alcoholic fermentation are not only the consumption of sugar and the synthesis of ethanol, but also the production of other secondary metabolites such as glycerol. At the end of alcoholic fermentation, the residual sugar content in both wines was less than 4 g/L, indicating that both *S. cerevisiae* completed the fermentation of Prince dry red wine. The glycerol content in the wine inoculated with strain L59 was 8.81 ± 0.06 g/L, which was significantly higher than the 8.31 ± 0.14 g/L in the wine inoculated with yeast FX10. It has been proved that the sensory threshold of glycerol in dry red wine is 7.5 g/L [36]. Glycerol above sensory thresholds in both dry red wines improved the perception of sweetness. At the same time, glycerol can increase perceived viscosity, thereby increasing the roundness of the wine tasting, and it can also suppress unpleasant mouthfeel such as heat, harshness and bitterness brought by ethanol [37]. Organic acids in wine are also a kind of metabolites, which can be obtained directly from grape berries or produced by microbial metabolism [38]. Non-volatile organic acids are the main contributors to the perception of acidity in wines and are one of the indicators to measure the balance of wine taste [39]. Among them, tartaric acid is one of the contributors to the sour taste of wine. The tartaric acid concentrations in the L59 wine and the FX10 wine were 4.73 ± 0.08 g/L and 5.09 ± 0.10 g/L, respectively. However, high concentrations (>5 g/L) of tartaric acid can cause strong unpleasant acidity in wines [40]. Malic acid is a green and astringent acid that can be degraded by yeast through malo-ethanolic fermentation, in which malic acid is converted to pyruvate under the catalysis of malic enzyme [41]. The previous study has shown that commercial *S. cerevisiae* has lower malic enzyme activity than indigenous yeast [42]. Similarly, in this study, commercial *S. cerevisiae* FX10 degraded 12.59% of malic acid in Prince grape must, while indigenous *S. cerevisiae* L59 degraded 28.67%. The degradation efficiency of malic acid in the L59 group was significantly higher than that in the FX10 group, which weakened the green and astringency contributed by malic acid. Besides, succinic acid also presents a salty and bitter taste in wine [43]. Additionally, citric acid not only contributes acidity to the wine, but also brings a pleasant freshness perception [40,44].

Along with alcoholic fermentation, many volatile compounds are produced by microorganisms, and these volatile components are closely related to wine aroma. Bokulich et al. [34] proved that the metabolites in wine were correlated with the species and abundance of wine-fermenting microorganisms based on microbial community analysis and the determination of volatile compounds in wine. Our results are consistent with previous findings, demonstrating that the composition and abundance of the fungal community can affect the aroma quality of wine [45]. Compared with grape must, the volatile compounds in wines had been greatly changed due to the metabolic activity of fungi. In this study, *Saccharomyces, Hanseniaspora, Starmerella, Botrytis, Cladosporium, Torulaspora, Zygosaccharomyces and unclassified_f__Nectriaceae* were defined as the core fungi involved in the fermentation of two groups of Prince wine. Their abundance changes were significantly correlated with the production of volatile compounds such as alcohols, esters, and acids. *S. cerevisiae* and other fungal taxa showed a completely different pattern in the correlation of volatile compounds. Among them, *S. cerevisiae* was positively correlated with most alcohols and esters. *S. cerevisiae* is the dominant player in the alcoholic fermentation of wine and is the main producer of alcohols and esters [46]. However, the production of volatile compounds is associated with more than one microorganism. The extracellular enzymes secreted by fungi other than *S. cerevisiae*, such as β-glucosidase, protease, and esterase, can also change the complexity of wine aroma [47]. The different correlations of fungal communities in the two groups of fermentations affected the flavor profile of the wines. The different correlations of fungal communities in the two groups of fermentations affected the flavor profile of the wines. Compared with commercial *S. cerevisiae* FX10, inoculation of indigenous *S. cerevisiae* L59 for wine fermentation was more beneficial to preserve the varietal aroma characteristics of the grape. The influence of microorganisms on the synthesis and metabolism of volatile compounds has been studied to the species level or strain level [48,49,50]. Therefore, the impact of fungal microorganisms on the production of volatile compounds needs to be further evaluated.

## 4. Materials and Methods

### 4.1. Grape Variety

The Prince grape is a hybrid (Chambourcin × Viognier) wine grape variety with dark purple skin. The Prince grapes harvested in September 2021 contained 248.78 g/L total sugar and 7.36 g/L total acid (as tartaric acid).

### 4.2. Interdelta Sequence Typing

The TY1 retrotransposon region of 5 commercial *S. cerevisiae* strains and the indigenous *S. cerevisiae* L59 isolated from winery was amplified using the interdelta fingerprinting method. PCR amplification was performed with primers delta12 (5′-TCAACAATGGAATCCCAAC-3′) and delta21 (5′-CATCTTAACACCGTATATGA-3′) [51]. The PCR reaction volume was 30 µL, containing 15 µL 2× M5 HiPer plus Taq HiFi PCR mix (Mei5 Biotechnology, Co., Ltd., Beijing, China), 0.75 µL forward primer (10 µM), 0.75 µL reverse primer (10 µM), 3 µL template DNA and double-distilled water. The PCR cycling conditions were as follows: 95 °C pre-degeneration for 4 min; 95 °C degeneration for 30 s, 46 °C annealing for 30 s, 72 °C extension for 90 s, repeated 35 times; complementary extension at 72 °C for 10 min. The amplified products were separated by electrophoresis on 1% agarose gel submitted to 120 V (constant voltage) in 1× TAE buffer, and photographed under UV light.

### 4.3. Winemaking and Sampling

The Prince grapes were divided into two fermentation tanks after destemming, crushing, and pressing. Pectinase (0.02 g/L, Lafase He Grand Cru, Laffort, France), potassium metabisulfite (0.075 g/L, Potassium metabisulfite, Laffort, France), and tannin (0.2 g/L, Tanin Vr Color, Laffort, France) were added to both tanks. One group was inoculated with the active dry yeast L59 (0.2 g/L, indigenous *S. cerevisiae*) and the other group was inoculated with the commercial active dry yeast (0.2 g/L, Zymaflore FX10, Laffort, France). Fermentations were all carried out in 1 m^3^ tanks (fermentation volume 800 L). The must was agitated by pump twice a day during fermentation and 45 mL fermentation samples were collected in sterile centrifuge tubes in triplicate after cycling. When the residual sugar content was lower than 4 g/L and no carbon dioxide bubbles was found, the alcoholic fermentation was considered finished. All samples were stored at −20 °C immediately after sampling in the winery. Samples collected on days 0, 2, 3, 4, and 10 of alcoholic fermentation were subjected to high-throughput sequencing (HTS) and stored at −80 °C.

### 4.4. DNA Extraction and Sequencing

The total DNA in the samples was extracted using the FastDNA Spin Kit (MP Biomedicals, Santa Ana, CA, USA). The integrity of DNA was checked by using a 1% agarose gel at a voltage of 5 V/cm for 20 minutes. The internal transcribed spacer (ITS) region was amplified using forward primer ITS1F (5′-CTTGGTCATTTAGAGGAAGTAA-3′) and reverse primer ITS2R (5′-GCTGCGTTCTTCATCGATGC-3′). PCR amplification of each sample (20 µL reaction volume) included 10 µL 2× Pro Taq Master Mix (Accurate Biotechnology (Hunan) Co., Ltd., Hunan, China), 0.8 µL forward primer (5 µM), 0.8 µL reverse primer (5 µM), 10 ng template DNA and double-distilled water. The PCR cycling conditions were as follows: 95 °C pre-degeneration for 3 min; 95 °C degeneration for 30 s, 55 °C annealing for 30 s, 72 °C extension for 45 s, repeated 35 times; complementary extension at 72 °C for 10 min. The PCR products were detected via 2% agarose gel electrophoresis and then purified using the AxyPrep DNA Gel Extraction Kit (AXYGEN Biosciences, San Francisco, CA, USA). The purified PCR products were submitted to Majorbio Bio-Pharm Technology Co., Ltd., (Shanghai, China) for paired-end sequencing of the ITS region based on the Illumina Miseq platform. The raw sequencing reads in FASTQ format were processed using QIIME software (version 1.9.1) to control sequence quality and filter low-quality sequences. Using Flash software (version 1.2.11, ) (Magoc and Salzberg 2011) (California, USA), according to the overlap relationship between PE reads, the paired reads were merged into a sequence with a minimum overlap length of 10bp. Sequences with 97% similarity were clustered into Operation Taxonomic Units (OTU) by using Usearch software (version 11, Edgar, http://www.drive5.com/usearch/ accessed on 10 September 2022). The most abundant sequences in each OTU were selected as representative sequences. OTU sequences were annotated by comparison with UNITE (version 8.0) database. To avoid or reduce the impact of different sequencing depths on the results, the OTU abundances were normalized using the fewest number of sequences in the samples to obtain normalized data. Data collection was done on the Majorbio cloud platform.

### 4.5. Physicochemical Parameters Analysis

The contents of sugars, organic acids, ethanol and glycerol were determined by HPLC. The chromatographic conditions for the determination of glucose, fructose, glycerol and ethanol in the samples referred to the method of Sun et al. [52]: refractive index detector (Waters-2414, Waters Corp., Dublin, Ireland), internal temperature 40 °C, Aminex HPX-87H column (300 mm × 7.8 mm), column temperature 55 °C, mobile phase 0.005 mol/L H_2_SO_4_, flow rate 0.5 mL/min, injection volume 10 µL, isocratic elution. The chromatographic conditions for the determination of organic acids in the samples were based on the method of Gao et al. [53] with minor modifications: photodiode array detector (Waters-2996, Waters Corp., Dublin, Ireland), detection wavelength 210 nm, Aminex HPX-87H column (300 mm × 7.8 mm), column temperature 60 °C, mobile phase 0.005 mol/L H_2_SO_4_, flow rate 0.6 mL/min, injection volume 10 µL, isocratic elution. Compounds were qualified and quantified based on retention time and peak area.

### 4.6. Volatile Compounds Analysis

The volatile compounds in the samples were analyzed by HS-SPME-GC-MS (Agilent, Santa Clara, CA, USA), referring to the method of Chen et al. [54] with modifications. Each vial contained 5 mL of sample, 2 g of NaCl, and 10 µL of 2-octanol (100 mg/mL, Sigma-Aldrich, St. Louis, MI, USA). The vials were incubated for 15 min at 60 °C, 250 rpm, shaking for 10 s, and stopping for 10 s. After incubation, the DVB/CAR/PDMS fiber (50/30 µm, ANPEL Corp., Shanghai, China) was inserted into the headspace of the vial to adsorb the volatile compounds in the sample for 30 min. The fiber was then inserted into the injection port (250 °C) for 5 min of desorption and separated on the HP-INNOWAX column (30m × 0.25 mm × 0.25 µm, J&W Scientific Agilent Technologies, Palo Alto, CA, USA) in constant flow mode (1.0 mL/min). The oven temperature program was as follows: the initial temperature was kept at 35 °C for 5 min, increased to 45 °C at a rate of 2 °C/min, then increased to 180 °C at a speed of 3 °C/min, and finally increased to 230 °C at a speed of 10 °C/min and held for 5 min. The signals were collected in full scan mode (29–500 amu) via electron ionization (EI) at 70 eV. Additionally, the temperatures of the MS interface, ion source, and quadrupole were 280 °C, 230 °C, and 150 °C. Volatile compounds were identified by comparison with the NIST 14 database, and the contents were semi-quantified by the internal standard (2-octanol). All samples were tested in triplicate.

### 4.7. Statistical Analysis

Three parallel samples of all sample groups were analyzed, and the results were expressed as mean ± standard deviation (SD). SPSS 25.0 software (SPSS Inc., Chicago, IL, USA) was used for one-way analysis of variance (ANOVA, significance level was 0.05) based on Tukey test or Duncan test. The Shapiro–Wilk’s and Levene’s tests were used to test the normality and homogeneity of variances. Principal component analysis (PCA) was performed using origin 2019b software (OriginLab, Northampton, MA, USA). Spearman’s correlation analysis was used to analyze the correlation between microbial species and volatile compounds, which was visualized using R (version 4.1.2, Robert Gentleman and Ross Ihaka, Auckland, New Zealand).

## 5. Conclusions

This study revealed the differences in the perturbation of natural fungal community in grape must and the improvement of wine aroma when indigenous *S. cerevisiae* L59 and commercial *S. cerevisiae* FX10 were applied to ferment Prince wine. Compared with commercial *S. cerevisiae* FX10, indigenous *S. cerevisiae* L59 could better adapt to the physicochemical conditions and natural microbial ecology of grape must. Moreover, when indigenous *S. cerevisiae* L59 was applied to the wine fermentation of the grape from the same production region, it was beneficial to improving the flavor and aroma quality of the wine, and was more conducive to retaining the aroma characteristics of local grape varieties.

## Figures and Tables

**Table 1:** The physicochemical indexes of Prince grape must and wine.

	Grape Must (Day 0)	Wine	
		L59	FX10
Glucose (g/L)	115.59 ± 0.05a	0.64 ± 0.02b	0.65 ± 0.00b
Fructose (g/L)	133.19 ± 0.16a	1.42 ± 0.03b	1.44 ± 0.05b
Glycerol (g/L)	N.D.	8.81 ± 0.06a	8.31 ± 0.14b
Ethanol (% *v*/*v*)	N.D.	13.10 ± 0.04	13.46 ± 0.21
Citric acid (g/L)	0.59 ± 0.01c	1.14 ± 0.01a	1.11 ± 0.01b
Tartaric acid (g/L)	7.36 ± 0.09a	4.73 ± 0.08c	5.09 ± 0.10b
Malic acid (g/L)	1.43 ± 0.01a	1.02 ± 0.01c	1.25 ± 0.02b
Succinic acid (g/L)	0.55 ± 0.02b	1.39 ± 0.04a	1.37 ± 0.04a
Lactic acid (g/L)	N.D.	0.73 ± 0.01	0.73 ± 0.02
Acetic acid (g/L)	0.19 ± 0.00c	0.29 ± 0.01b	0.34 ± 0.02a

Note: Different letters in the same row indicate that the content levels of the measured substances are significantly different, and unmarked letters indicate that there is no significant difference, *p* < 0.05. N.D. denotes not detected.

## Data Availability

Not applicable.

## Supplementary Materials

The following supporting information can be downloaded at: https://www.mdpi.com/article/10.3390/molecules27206892/s1, Figure S1: Heatmap cluster analysis of volatile compounds during fermentation of Prince wine; Figure S2: Correlations between microorganisms and volatile compounds analyzed by O2PLS modeling. Plot of VIP scores for fungi and volatile compounds in L59 (a) and FX10 (b); Table S1: Concentrations of volatile compounds during fermentation [55,56,57,58,59,60,61,62,63,64,65,66]; Table S2: Alpha diversity of fungal community during Prince fermentation.

## Sample Availability

Samples of the compounds are not available from the authors.
