# Supplementary material for: Indigenous Saccharomyces cerevisiae Could Better Adapt to the Physicochemical Conditions and Natural Microbial Ecology of Prince Grape Must Compared with Commercial Saccharomyces cerevisiae FX10
Source: Molecules. 2022 Oct 14;27(20):6892. doi: 10.3390/molecules27206892 (PMC9610378; doi:10.3390/molecules27206892)
Supplement: Supplementary file 1 [file molecules-27-06892-s001.zip › molecules-1954033-supplementary.pdf]

Table S1. Concentrations of volatile compounds during fermentation.

Category	Compounds	Number	Odor threshold (μg/L)[1]	Concentrations (μg/L)										
				Grape Must	L59					FX10				
					D2	D3	D4	D10	OAV	D2	D3	D4	D10	OAV
Alcohols	Phenethyl alcohol	V1	14000 [55]	105.33 ± 9.94cd	141.93 ± 18.13cd	343.97 ± 16.55bcd	379.12 ± 9.25bc	6000.32 ± 196.10a	0.43	79.69 ± 3.46d	235.61 ± 20.40cd	604.77 ± 18.90b	6192.04 ± 127.77a	0.44
	2-Methyl-1-propanol	V2	75000 [56]	168.74 ± 1.54abc	227.76 ± 99.94ab	111.42 ± 6.11bcd	84.32 ± 23.99cd	N.D.		107.05 ± 12.88bcd	258.59 ± 31.40a	110.73 ± 20.35bcd	N.D.	
	2-Ethylhexanol	V3		61.09 ± 2.47c	14.37 ± 1.40d	33.86 ± 0.93cd	40.33 ± 1.68cd	487.43 ± 21.35a		13.05 ± 0.13d	21.56 ± 0.35cd	27.15 ± 1.59cd	333.06 ± 30.06b	
	Hexyl alcohol	V4	1100 [56]	2246.88 ± 225.30a	537.21 ± 75.18c	150.05 ± 11.19d	98.08 ± 3.80d	1174.14 ± 75.03b	1.07	645.43 ± 12.96c	184.27 ± 5.86d	102.09 ± 3.32d	1222.40 ± 26.38b	1.11
	1-Octanol	V5	800 [16]	11.96 ± 1.08b	3.93 ± 0.56b	12.88 ± 0.74b	14.23 ± 0.99b	98.82 ± 8.82a	0.12	2.81 ± 0.15b	9.94 ± 0.85b	14.62 ± 0.94b	95.34 ± 10.06a	0.12
	3-Methyl-1-butanol	V6	40000 [57]	936.65 ± 53.87b	N.D.	2041.05 ± 118.52b	2024.06 ± 498.49b	51556.49 ± 1706.02a	1.29	N.D.	1929.14 ± 245.61b	3165.15 ± 73.57b	52249.37 ± 1326.41a	1.31
	(E)-2-Hexen-1-ol	V7	400 [55]	72.09 ± 10.63a	6.78 ± 0.80b	N.D.	N.D.	N.D.		5.66 ± 0.14b	N.D.	N.D.	N.D.	
	Leaf alcohol	V8	1000 [56]	78.01 ± 3.24a	23.10 ± 1.50b	N.D.	N.D.	N.D.		20.18 ± 0.82b	5.64 ± 0.67c	N.D.	N.D.	
	Benzyl alcohol	V9	200000 [56]	5.54 ± 0.12b	2.28 ± 0.40b	2.90 ± 0.06b	2.24 ± 0.13b	54.94 ± 18.34a	0.00	2.83 ± 0.14b	2.60 ± 0.31b	3.66 ± 0.21b	N.D.	
	3-Methylthiopropanol	V10	1000 [55]	20.41 ±	18.92 ±	19.89 ±	14.71 ±	172.29 ±	0.17	11.50 ±	24.27 ±	26.66 ±	447.75 ±	0.45

Category	Compounds	Number	Odor threshold (μg/L)[1]	Concentrations (μg/L)										
				Grape Must	L59					FX10				
					D2	D3	D4	D10	OAV	D2	D3	D4	D10	OAV
				12.21c	3.05c	1.64c	0.59c	3.44b		0.80c	1.81c	1.75c	49.50a	
	(Z)-Linalool oxide	V11	500 [58]	12.62 ± 7.92	N.D.	N.D.	N.D.	N.D.		N.D.	N.D.	N.D.	N.D.	
	1-Pentanol	V12	64000 [59]	N.D.	N.D.	N.D.	N.D.	52.28 ± 10.52a	0.00	9.73 ± 1.51b	N.D.	N.D.	48.87 ± 19.47ab	0.00
	3-Ethoxy-1-propanol	V13	100 [60]	N.D.	5.83 ± 0.66b	8.11 ± 0.82b	5.01 ± 0.47b	70.87 ± 2.52a	0.71	3.21 ± 0.38b	7.15 ± 0.20b	6.45 ± 0.60b	59.52 ± 11.23a	0.60
	2,3-Butanediol	V14	150000 [59]	N.D.	N.D.	105.94 ± 16.08b	136.88 ± 55.57b	2458.19 ± 136.28a	0.02	N.D.	35.61 ± 10.63b	107.92 ± 23.61b	2260.69 ± 283.67a	0.02
	4-Methyl-1-pentanol	V15	50000 [55]	N.D.	N.D.	N.D.	N.D.	55.52 ± 8.73	0.00	N.D.	N.D.	N.D.	66.75 ± 11.49	0.00
	1-Butanol	V16	15000 [56]	N.D.	11.11 ± 7.14	N.D.	14.88 ± 2.54	N.D.		10.99 ± 1.26	N.D.	13.53 ± 2.33	N.D.	
	(Z)-2-Hexen-1-ol	V17	400 [61]	N.D.	3.65 ± 0.35	N.D.	N.D.	N.D.		3.61 ± 0.26	N.D.	N.D.	N.D.	
	1-Propanol	V18	306000 [56]	N.D.	N.D.	N.D.	32.99 ± 14.14	N.D.		N.D.	N.D.	34.20 ± 1.53	N.D.	
Acids	Isobutyric acid	V19	2300 [55]	19.27 ± 1.53c	17.39 ± 2.91c	N.D.	N.D.	106.16 ± 16.14b	0.05	26.94 ± 0.27c	19.10 ± 1.56c	N.D.	210.54 ± 43.22a	0.09
	Octanoic acid	V20	500 [55]	2.73 ± 0.48c	16.57 ± 2.81c	54.91 ± 0.39c	32.11 ± 5.13c	843.50 ± 7.30b	1.69	18.99 ± 1.14c	71.42 ± 4.93c	69.63 ± 6.87c	1386.28 ± 82.03a	2.77
	Hexanoic acid	V21	420 [55]	73.71 ± 5.56c	32.12 ± 4.76c	36.74 ± 0.99c	19.91 ± 1.61c	518.92 ± 82.30b	1.24	29.64 ± 1.82c	44.87 ± 4.07c	42.81 ± 1.60c	787.36 ± 28.53a	1.87

Category	Compounds	Number	Odor threshold (μg/L) [1]	Concentrations (μg/L)										
				Grape Must	L59					FX10				
					D2	D3	D4	D10	OAV	D2	D3	D4	D10	OAV
	(E)-2-Hexenoic acid	V22		40.63 ± 2.97a	1.61 ± 0.30b	1.15 ± 0.17b	N.D.	N.D.		3.48 ± 0.16b	0.96 ± 0.08b	N.D.	N.D.	
	Decanoic acid	V23	1000 [46]	N.D.	2.64 ± 0.24c	11.81 ± 1.11b	10.43 ± 2.35b	N.D.		2.97 ± 0.60c	17.72 ± 2.43a	12.67 ± 2.27ab	N.D.	
Esters	Dibutyl phthalate	V24		27.51 ± 2.54b	6.46 ± 0.28b	4.49 ± 0.25b	7.10 ± 0.61b	75.01 ± 10.98a		12.86 ± 4.34b	6.01 ± 1.29b	6.51 ± 1.35b	66.53 ± 19.17a	
	Ethyl caprate	V25	200 [62]	21.72 ± 4.41e	59.04 ± 7.48de	679.28 ± 33.94cd	1312.85 ± 9.48bc	2646.19 ± 449.33a	13.23	19.33 ± 0.90e	314.25 ± 18.74de	1542.70 ± 313.59b	1892.86 ± 78.04b	9.46
	Diethyl succinate	V26	200000 [57]	6.72 ± 2.47d	5.69 ± 0.74d	34.45 ± 2.01bcd	45.96 ± 1.34bc	121.28 ± 1.50a	0.00	5.28 ± 0.08d	26.66 ± 0.63cd	62.12 ± 0.49b	103.66 ± 28.40a	0.00
	Methyl 14-methylpentadecanoate	V27		48.81 ± 63.52	N.D.	N.D.	N.D.	N.D.		N.D.	N.D.	N.D.	N.D.	
	Ethyl lactate	V28	14000 [57]	N.D.	5.56 ± 0.68b	9.08 ± 0.63b	11.27 ± 0.80b	164.05 ± 38.42a	0.01	N.D.	6.01 ± 0. 06b	11.25 ± 0.29b	151.47 ± 25.53a	0.01
	Ethyl caprylate	V29	580 [56]	N.D.	229.98 ± 26.23e	1492.31 ± 53.55de	2601.80 ± 143.83cd	10115.04 ± 347.20a	17.44	44.79 ± 0.68e	927.87 ± 49.94e	3308.78 ± 532.78c	7496.70 ± 1144.72b	12.93
	Ethyl laurate	V30	1500 [62]	N.D.	N.D.	131.17 ± 18.03c	236.64 ± 18.93bc	512.49 ± 112.93a	0.34	N.D.	84.37 ± 6.28c	345.04 ± 78.56ab	500.83 ± 20.82a	0.33
	Methyl octanoate	V31	200 [63]	N.D.	33.73 ± 3.39c	103.27 ± 1.64b	74.48 ± 3.87b	163.89 ± 12.30a	0.82	19.77 ± 1.59c	162.06 ± 7.03a	90.14 ± 9.72b	172.00 ± 17.89a	0.86
	Methyl hexadecanoate	V32		N.D.	1.08 ± 0.28b	3.40 ± 0.57b	2.18 ± 0.19b	21.39 ± 5.90a		1.64 ± 0.24b	3.04 ± 0.67b	3.26 ± 0.48b	23.58 ± 12.62a	
	Methyl salicylate	V33	100 [62]	N.D.	3.79 ±	6.91 ±	4.42 ±	51.02 ±	0.51	4.38 ±	7.05 ±	8.26 ±	N.D.	

Category	Compounds	Number	Odor threshold (μg/L)[1]	Concentrations (μg/L)										
				Grape Must	L59					FX10				
					D2	D3	D4	D10	OAV	D2	D3	D4	D10	OAV
					0.56b	0.81b	0.56b	9.78a		0.18b	0.50b	0.81b		
	Isoamyl acetate	V34	160 [56]	N.D.	261.58 ± 49.50b	371.47 ± 28.72b	735.64 ± 82.62b	11579.30 ± 265.22a	72.37	151.12 ± 20.18b	360.75 ± 6.40b	911.87 ± 71.45b	12463.22 ± 1638.76a	77.90
	Ethyl myristate	V35	800 [57]	N.D.	0.84 ± 0.13d	20.97 ± 3.22cd	31.18 ± 0.77cd	94.51 ± 21.97a	0.12	0.52 ± 0.02d	17.66 ± 1.82cd	44.91 ± 5.81bc	77.88 ± 19.97ab	0.10
	Hexyl acetate	V36	1500 [55]	N.D.	19.48 ± 2.41c	19.48 ± 0.50c	29.33 ± 0.65c	187.60 ± 18.27b	0.13	14.87 ± 0.44c	21.17 ± 1.63c	52.52 ± 5.31c	265.76 ± 47.37a	0.18
	Ethyl palmitate	V37	1500 [57]	N.D.	2.10 ± 0.12b	39.37 ± 7.25b	40.15 ± 1.50b	323.62 ± 135.12a	0.22	1.84 ± 0.21b	20.77 ± 3.56b	63.70 ± 5.35b	306.41 ± 27.51a	0.20
	Ethyl linolenate	V38		N.D.	3.12 ± 0.32b	7.42 ± 0.37b	6.03 ± 0.37b	66.09 ± 9.98a		6.57 ± 2.05b	9.34 ± 1.84b	7.81 ± 1.37b	81.32 ± 22.30a	
	3-methylbutyl octanoate	V39	125 [55]	N.D.	N.D.	14.04 ± 1.50c	20.83 ± 0.49bc	58.46 ± 13.95a	0.47	N.D.	8.90 ± 0.55c	20.57 ± 0.91bc	44.86 ± 10.82ab	0.36
	9-Hexadecenoic acid,ethyl ester	V40		N.D.	2.37 ± 0.23c	11.11 ± 1.60c	16.48 ± 2.61c	173.58 ± 17.56a		1.71 ± 0.06c	8.10 ± 1.20c	14.46 ± 2.66c	80.69 ± 1.62b	
	Ethyl 9-decenoate	V41	100 [61]	N.D.	N.D.	8.54 ± 1.22c	45.35 ± 1.33b	118.95 ± 5.43a	1.19	N.D.	2.85 ± 0.23c	71.23 ± 14.42b	110.34 ± 15.20a	1.10
	Ethyl octadecanoate	V42	15000 [64]	N.D.	N.D.	1.03 ± 0.19b	1.13 ± 0.12b	40.00 ± 8.25a	0.00	N.D.	1.34 ± 0.47b	1.33 ± 0.26b	N.D.	
	Ethyl oleate	V43		N.D.	1.32 ± 0.35b.	4.21 ± 0.45b	4.94 ± 1.04b	73.13 ± 13.08a		1.96 ± 0.57b	4.53 ± 0.92b	6.41 ± 2.27b	11.04 ± 1.79b	
	Diisobutyl phthalate	V44		N.D.	2.71 ±	N.D.	N.D.	N.D.		3.74 ±	2.51 ±	N.D.	N.D.	

Category	Compounds	Number	Odor threshold (μg/L)[1]	Concentrations (μg/L)										
				Grape Must	L59					FX10				
					D2	D3	D4	D10	OAV	D2	D3	D4	D10	OAV
					0.51					0.65	0.39			
	Phenethyl acetate	V45	250 [65]	N.D.	58.45 ± 6.06ab	72.11 ± 2.62ab	130.83 ± 70.92a	N.D.		25.96 ± 0.35b	45.37 ± 0.88ab	N.D.	N.D.	
	Ethyl heptanoate	V46	300 [46]	N.D.	7.99 ± 1.24bc	4.79 ± 2.17c	15.88 ± 0.18a	N.D.		N.D.	7.87 ± 0.43bc	12.46 ± 2.45ab	N.D.	
	Methyl hexanoate	V47		N.D.	15.74 ± 1.46ab	14.47 ± 0.17b	10.52 ± 1.04b	N.D.		10.16 ± 0.39b	22.87 ± 5.52a	11.13 ± 1.43b	N.D.	
	Methyl n-caprate	V48	50 [46]	N.D.	12.91 ± 1.68b	51.98 ± 5.04a	46.59 ± 2.77a	N.D.		6.25 ± 0.43b	52.84 ± 4.76a	51.86 ± 7.43a	N.D.	
	Isopentyl formate	V49		N.D.	1941.98 ± 342.16	N.D.	N.D.	N.D.		N.D.	N.D.	N.D.	N.D.	
	Methyl laurate	V50		N.D.	4.06 ± 0.48a	13.36 ± 1.61b	11.33 ± 1.79b	N.D.		N.D.	11.96 ± 1.27b	13.37 ± 2.75b	N.D.	
	Ethyl caproate	V51	14 [57]	N.D.	205.62 ± 19.24d	826.80 ± 48.31bc	976.69 ± 64.49b	N.D.		55.13 ± 0.30d	769.22 ± 22.96c	1340.92 ± 84.79a	N.D.	
	Diisobutyl adipate	V52		N.D.	1.39 ± 0.10bc	1.30 ± 0.11bc	1.74 ± 0.10b	N.D.		2.59 ± 0.32a	0.99 ± 0.03c	N.D.	N.D.	
	Ethyl 3-phenylpropionate	V53		N.D.	1.25 ± 0.19d	6.67 ± 0.24bc	7.60 ± 0.09b	N.D.		N.D.	5.90 ± 0.34c	11.17 ± 1.03a	N.D.	
	2-Ethylhexyl acetate	V54		N.D.	N.D.	3.99 ± 0.73b	9.15 ± 1.25a	N.D.		N.D.	N.D.	10.53 ± 1.05a	N.D.	
	Ethyl nonanoate	V55	1300 [57]	N.D.	N.D.	2.40 ± 0.69b	9.68 ± 0.52a	N.D.		N.D.	N.D.	2.40 ± 0.46b	N.D.	

Category	Compounds	Number	Odor threshold (μg/L)[1]	Concentrations (μg/L)										
				Grape Must	L59					FX10				
					D2	D3	D4	D10	OAV	D2	D3	D4	D10	OAV
	Methyl tetradecanoate	V56		N.D.	N.D.	2.93 ± 1.09	1.71 ± 0.28	N.D.		N.D.	2.97 ± 0.32	1.53 ± 0.26	N.D.	
	Decanoic acid, 3-methylbutyl ester	V57		N.D.	N.D.	6.43 ± 0.48bc	10.54 ± 0.79ab	N.D.		N.D.	5.15 ± 0.55c	15.12 ± 2.87a	N.D.	
	Ethyl (E)-hex-2-enoate	V58		N.D.	N.D.	2.21 ± 0.19	N.D.	N.D.		N.D.	N.D.	N.D.	N.D.	
	Isopentyl hexanoate	V59	1000 [46]	N.D.	N.D.	N.D.	19.23 ± 1.34a	N.D.		N.D.	6.82 ± 0.97b	N.D.	N.D.	
	Isobutyl decanoate	V60		N.D.	N.D.	N.D.	1.92 ± 0.48ab	N.D.		N.D.	1.10 ± 0.17b	3.11 ± 0.71a	N.D.	
	Ethyl linoleate	V61		N.D.	N.D.	12.06 ± 2.90b	13.36 ± 1.68b	166.32 ± 18.31a		N.D.	12.20 ± 0.43b	13.54 ± 2.33b	132.65 ± 21.22a	
	Octyl acetate	V62	50000 [57]	N.D.	N.D.	N.D.	3.06 ± 0.45	N.D.		N.D.	N.D.	5.15 ± 1.01	N.D.	
	Ethyl undecanoate	V63	100 [57]	N.D.	N.D.	N.D.	0.72 ± 0.21	N.D.		N.D.	N.D.	1.20 ± 0.23	N.D.	
	2-Phenylethyl caprylate	V64		N.D.	N.D.	N.D.	1.58 ± 0.22	N.D.		N.D.	N.D.	1.42 ± 0.30	N.D.	
	Isoamyl laurate	V65		N.D.	N.D.	N.D.	4.26 ± 0.92b	N.D.		N.D.	N.D.	9.94 ± 1.38a	N.D.	
Aldehydes and ketones	Benzaldehyde	V66	2000 [56]	35.97 ± 6.53a	35.57 ± 4.87a	9.73 ± 0.53b	7.60 ± 0.67b	N.D.		42.75 ± 0.78a	14.59 ± 1.74b	11.16 ± 0.45b	N.D.	
	2-Octanone	V67	250 [57]	25.54 ±	N.D.	N.D.	2.07 ±	N.D.		8.20 ±	N.D.	N.D.	N.D.	

Category	Compounds	Number	Odor threshold (μg/L)[1]	Concentrations (μg/L)										
				Grape Must	L59					FX10				
					D2	D3	D4	D10	OAV	D2	D3	D4	D10	OAV
				0.25a			0.11c			2.41b				
	(E E)-2,4-Hexadienal	V68		12.73 ± 0.47	N.D.	N.D.	N.D.	N.D.		N.D.	N.D.	N.D.	N.D.	
	2-Hexenal	V69	4 [61]	52.97 ± 2.22a	27.01 ± 1.69b	N.D.	N.D.	N.D.		N.D.	N.D.	N.D.	N.D.	
	3-Hydroxy-2-butanone	V70	150000 [57]	24.00 ± 0.87bc	37.64 ± 5.07a	22.11 ± 5.04bc	N.D.	N.D.		27.49 ± 3.22ab	15.04 ± 1.07c	3.74 ± 0.38d	N.D.	
	β-Damascenone	V71	0.05 [57]	7.67 ± 1.93	N.D.	N.D.	N.D.	N.D.		N.D.	N.D.	N.D.	N.D.	
	2-Nonanone	V72		14.08 ± 3.63	N.D.	N.D.	N.D.	N.D.		N.D.	N.D.	N.D.	N.D.	
	Hexanal	V73	20 [61]	N.D.	238.56 ± 27.30a	N.D.	N.D.	N.D.		177.61 ± 3.37b	29.55 ± 1.39c	N.D.	N.D.	
	4-Hydroxy-3-methylacetophenone	V74	1000000 [57]	N.D.	N.D.	N.D.	N.D.	N.D.		N.D.	1.07 ± 0.12	N.D.	N.D.	
Olefins	Phenylethylene	V75		18.41 ± 0.32c	64.24 ± 14.94bc	22.91 ± 2.41c	34.80 ± 1.50c	184.37 ± 67.78ab		N.D.	52.21 ± 3.26c	33.63 ± 0.69c	262.88 ± 84.08a	
	1,3,5,7-Cyclooctatetraene	V76		N.D.	N.D.	11.18 ± 8.25b	N.D.	N.D.		45.50 ± 6.75a	N.D.	N.D.	N.D.	
	Undecane	V77		N.D.	N.D.	N.D.	5.44 ± 2.58	N.D.		N.D.	N.D.	8.83 ± 0.88	N.D.	
	Benzene, 1,3-bis(1,1-dimethylethyl)-	V78		N.D.	N.D.	N.D.	N.D.	75.23 ± 8.01a		N.D.	N.D.	3.64 ± 0.27b	111.21 ± 33.13a	

Category	Compounds	Number	Odor threshold (μg/L)[1]	Concentrations (μg/L)										
				Grape Must	L59					FX10				
					D2	D3	D4	D10	OAV	D2	D3	D4	D10	OAV
Phenols	Naphthalene	V79		N.D.	N.D.	N.D.	1.99 ± 0.28	N.D.		N.D.	N.D.	N.D.	N.D.	
	α-Humulene	V80		N.D.	N.D.	N.D.	1.83 ± 0.29	N.D.		N.D.	N.D.	N.D.	N.D.	
	2,4-Di-tert-butylphenol	V81	200 [66]	23.39 ± 28.09b	0.94 ± 0.09b	1.76 ± 0.03b	1.00 ± 0.03b	155.74 ± 23.04a	0.78	1.80 ± 0.11b	1.68 ± 0.29b	1.23 ± 0.29b	197.07 ± 23.17a	0.99
	Phenol	V82		N.D.	1.00 ± 0.18	N.D.	N.D.	N.D.		1.32 ± 0.10	N.D.	N.D.	N.D.	
	Guaiacol	V83	9.5 [55]	N.D.	N.D.	N.D.	N.D.	N.D.		1.11 ± 0.18	N.D.	N.D.	N.D.	

Note: Different letters in the same row indicate that there are significant differences in the concentrations of volatile compounds, and those without letters indicate that there is no significant difference in the concentrations of volatile compounds, $p < 0.05$. N.D. denotes not detected. OAV (odor activity value) = volatile compound concentration/odor threshold.

[1]Odor thresholds were obtained from the literature.

Table S2. Alpha diversity of fungal community during Prince fermentation.

Samples	Shannon	Simpson	Sobs	Ace	Chao1	Coverage (%)
Grape Must	1.85 ± 0.12	0.36 ± 0.04	169.33 ± 4.99	215.80 ± 21.29	207.09 ± 13.69	1.00 ± 0.00
L59 2	1.57 ± 0.08	0.34 ± 0.03	97.00 ± 6.53	149.09 ± 24.70	131.22 ± 8.27	1.00 ± 0.00
FX10 2	1.44 ± 0.07	0.36 ± 0.02	102.33 ± 2.87	197.22 ± 43.30	174.69 ± 35.36	1.00 ± 0.00
L59 3	0.18 ± 0.06	0.95 ± 0.02	36.00 ± 11.05	86.34 ± 39.14	74.94 ± 38.53	1.00 ± 0.00
FX10 3	0.16 ± 0.03	0.96 ± 0.01	51.00 ± 7.79	103.33 ± 36.62	76.02 ± 20.23	1.00 ± 0.00
L59 4	0.13 ± 0.01	0.96 ± 0.00	37.67 ± 3.09	89.30 ± 26.57	65.79 ± 13.93	1.00 ± 0.00
FX10 4	0.06 ± 0.01	0.99 ± 0.00	31.00 ± 2.83	88.59 ± 33.79	51.54 ± 13.83	1.00 ± 0.00
L59 10	0.09 ± 0.03	0.97 ± 0.01	31.67 ± 3.68	64.37 ± 11.88	55.57 ± 12.26	1.00 ± 0.00
FX10 10	0.06 ± 0.01	0.98 ± 0.00	18.00 ± 6.48	65.36 ± 30.70	34.00 ± 14.61	1.00 ± 0.00

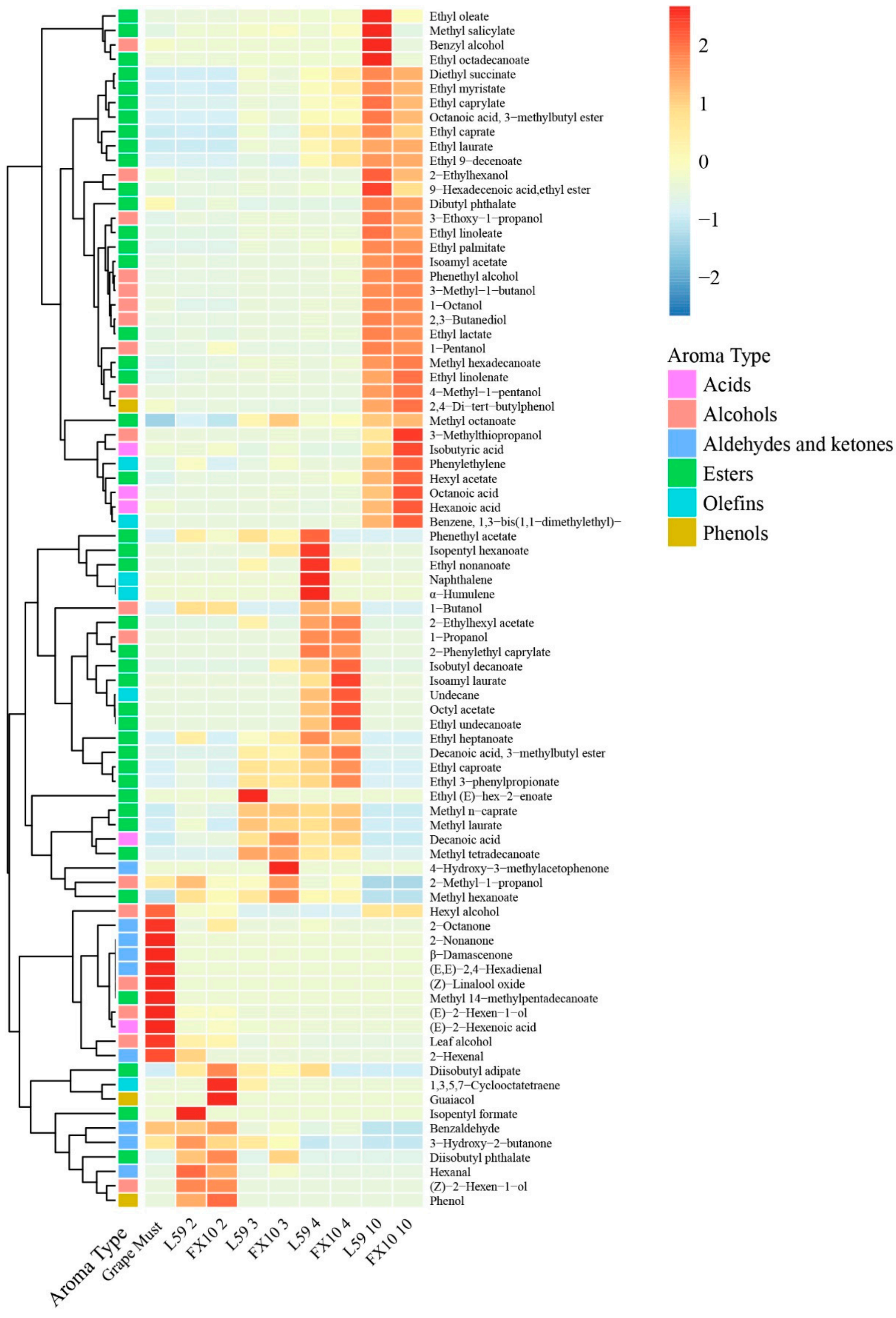

Figure S1. Heatmap cluster analysis of volatile compounds during fermentation of Prince wine.

(a)

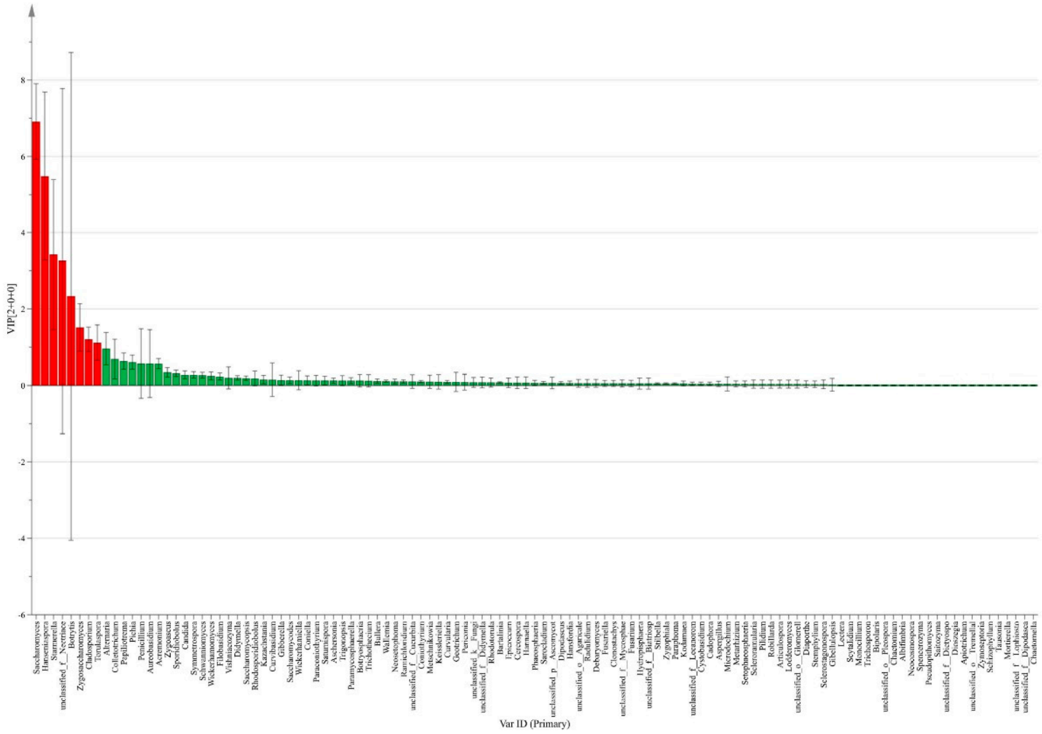

(b)

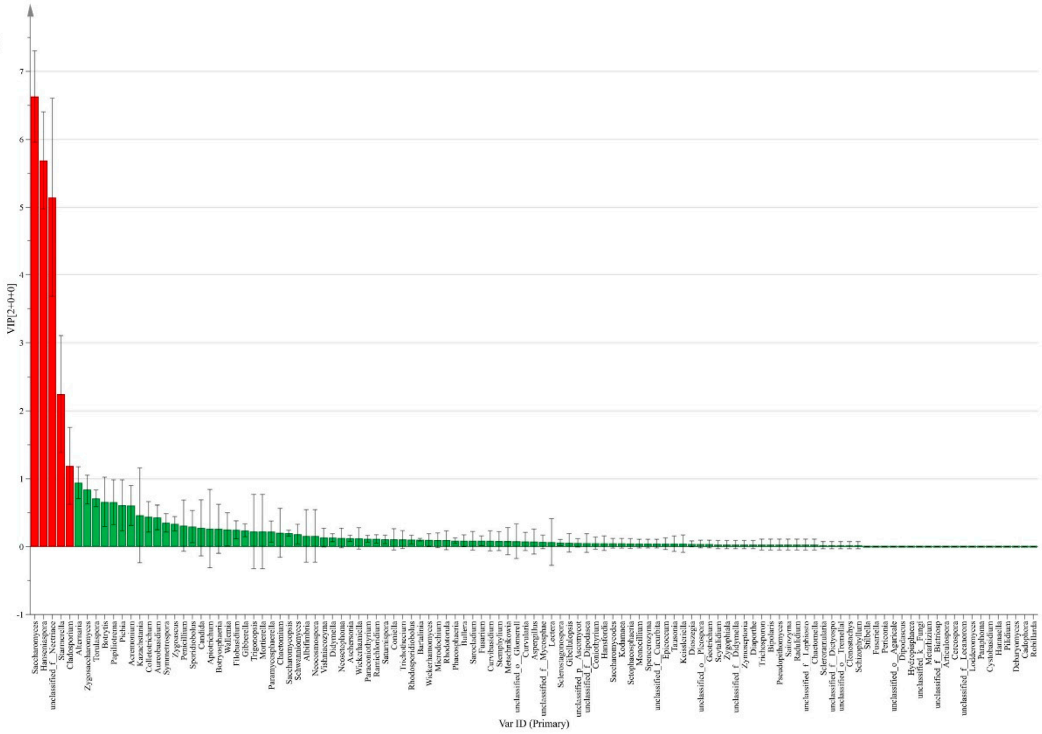

Figure S2. Correlations between microorganisms and volatile compounds analyzed by O2PLS modeling. Plot of VIP scores for fungi and volatile compounds in L59 (a) and FX10 (b). Note: Microorganisms with VIP > 1.0 were shown in red.
